# Low-frequency ultrasound-induced VEGF suppression and synergy with dendritic cell-mediated anti-tumor immunity in murine prostate cancer cells *in vitro*

**DOI:** 10.1038/s41598-017-06242-8

**Published:** 2017-07-18

**Authors:** Wei Zhang, Wen-De Shou, Yan-Jun Xu, Wen-Kun Bai, Bing Hu

**Affiliations:** Department of Ultrasound In Medicine, Shanghai Jiao Tong University Affiliated 6th People’s Hospital, Shanghai Institute of Ultrasound in Medicine, Shanghai, 200233 China

## Abstract

High tumor vascular endothelial growth factor (VEGF) levels are associated with poor treatment outcomes in prostate cancer (PCa), and immune deficiency in the PCa microenvironment, especially suppression of dendritic cell (DC) proliferation, has been confirmed. In this study, we (1) investigated whether VEGF participates in DC suppression in murine PCa cells (RM-1), (2) down-regulated VEGF expression using low-frequency ultrasound and microbubbles (UM), and (3) further explored any synergistic effect on immunological activation. DCs from the bone marrow of BALB/c mice were stimulated by the addition of cytokines (granulocyte-macrophage colony-stimulating factor (GM-CSF) and interleukin-4 (IL-4)), and we analyzed their proliferation status via flow cytometric recognition of the surface antigen markers CD11c and CD83. The results demonstrated that co-culture with RM-1 cells markedly inhibited expression of the general marker CD11c and the mature marker CD83; UM weakened this inhibition by down-regulating VEGF expression. T lymphocytes were extracted from murine spleens, and CD4 and CD8a were identified as the biomarkers of activated cells participating in the anti-tumor immune response. When DCs, T lymphocytes and RM-1 cells were co-cultured, cell migration and invasion assays and cytoactive detection showed that UM could not only directly suppress PCa cell evolution but also promote activation of anti-tumor immunocytes in the VEGF-inhibited microenvironment.

## Introduction

Prostate cancer (PCa) is the most common non-cutaneous cancer and the second leading cause of cancer-related death in the United States in recent years; it is also the most frequent cancer diagnosed in men in Europe^1^. Although most patients are diagnosed with organ-confined disease, for which radical prostatectomy and radiotherapy are effective treatment modalities, approximately 30% of patients develop recurrent disease^2^. Androgen-deprivation therapy (ADT) is the first-line gold standard for the treatment of advanced PCa^3–5^. However, despite initial response rates of 80–90%, the disease eventually progresses, and many patients develop metastatic castration-resistant PCa (mCRPC).

Dendritic cells (DCs) are the most powerful antigen-presenting cells (APCs) and may closely interact with tumor cells. Following exposure to tumor antigen, DCs migrate to peripheral lymph nodes and induce activation of cytotoxic T lymphocytes (CTLs) via antigen presentation; this process further triggers the immune response and induces immunological surveillance^6, 7^. DCs exhibit an extraordinary capacity to induce, sustain and regulate T lymphocyte responses, thus providing the possibility of DC-based cancer vaccination strategies^8^. As a result of various antitumor effects, DCs have emerged as promising candidates for the treatment of mCRPC patients and patients for whom local therapy is not appropriate. Consequently, several clinical trials based on the administration of DCs pulsed with tumor-associated antigens to PCa patients have been conducted^9, 10^. In addition, an autologous APC-based cancer vaccine, sipuleucel-T, was approved by the Food and Drug Administration (FDA) in 2010 and by the European Medicine Agency (EMA) in 2014 for the treatment of patients with asymptomatic or minimally symptomatic mCRPC^11^.

Vascular endothelial growth factor (VEGF), which induces neoangiogenesis and angiogenesis blockade, plays an important role in the development and metastasis of solid tumors, becoming a major target in cancer therapy^12^. Gallucci reported that suppression of VEGF in a mouse model leads to increased antigen uptake and migration of tumor-associated DCs^13^. Therefore, we speculated that inhibition of VEGF expression enhances DC differentiation and maturation in PCa, resulting in increased inhibition of tumorigenesis. It has been reported that the vascular endothelium is destroyed following treatment with ultrasound combined with a microbubble contrast agent (UCA)^14^; 1-MHz, low-intensity ultrasound also had an impact of fragile and leaky angiogenic blood vessels in tumors^15^. Our preliminary work confirmed that low-frequency ultrasound in combination with a contrast agent was effective for reducing expression of VEGF or COX-2 in the vascular endothelium and cytoplasm of PCa tumors^16^. In the present study, we down-regulated expression of VEGF in murine PCa cells using UCA and then co-cultured these cells with marrow-derived DCs and spleen-derived T lymphocytes to determine whether VEGF participates in the differentiation of immune cells. Furthermore, we investigated the migration, proliferation and metastasis ability of RM-1 cells to assess anti-tumor synergy of UCA-mediated angiogenesis destruction and immune cell activation.

## Methods

All experimental protocols were approved by the Institutional Review Board of the Shanghai Jiao Tong University Affiliated Sixth People’s Hospital (Shanghai, China). The methods involving animals were permitted by the ethics committee of Shanghai Jiao Tong University Affiliated Sixth People’s Hospital (Shanghai, China) and carried out in accordance with the standard guidelines of the Central Animal Facility of Shanghai Jiao Tong University Affiliated 6th People’s Hospital.

### Murine prostate cancer cells

The murine prostate cancer cell line RM-1 was obtained from the Cell Bank of the Chinese Academy of Science (Shanghai, China). The cells were cultured in RPMI-1640 (HyClone, Logan, UT, USA) supplemented with 10% heat-inactivated fetal bovine serum (FBS, Luoshen Biotechnology, Shanghai, China) at 37 °C in an incubator containing 5% CO_2_. For experiments, RM-1 cells were resuspended and counted to obtain a density of 10^5^ cells/ml; the cells were subsequently placed in 1.5-ml polystyrene sample test tubes. Each tube contained 1 ml of solution, and the tube diameter was 13 mm, which is in accordance with the ultrasound probe.

### Ultrasound installation and microbubbles

Inhibition of VEGF expression was achieved using an FY2200S ultrasonic instrument (Shanghai Institute of Ultrasound in Medicine, China), which includes a built-in digital timer, an intensity regulator and a duty factor controller. The transducer generated flat ultrasound frequency ranging from 20 kHz to 1 MHz; the duty cycle was fixed at 50% (0.5 sec “on” and 0.5 sec “off”) in all studies. The treatment mode used continuous waves. The spatial-average temporal average intensity (I_SATA_) ranged from 0.68 to 6.11 mW/mm^2^. The peak acoustic amplitude in degassed water was measured using a calibrated needle-type hydrophone that was 0.5 mm in diameter (Toray Techno Co., Ltd., Japan) and connected to a PC/AT-compatible computer as well as a digitizing oscilloscope (TDS3034, Tektronix Japan, Ltd., Japan). The shape of the probe was cylindrical, with a diameter of 13 mm, which was the same as the test tube diameter. In all experiments, the clamp was attached to a metal stand to ensure that the transducer faced directly upward. The tube was placed in the center of the transducer, which was intermediated with gel. This configuration enabled standing wave formation as a result of the reflection of ultrasound radiation at the water-air interface. We used a near acoustic field produced by the liquid-air interface and did not reduce the standing waves because of the efficient occurrence of cavitation^17, 18^.

SonoVue (Bracco SpA, Milan, Italy) comprises an ultrasound contrast agent with lipid-shelled, sulfur hexafluoride gas-filled microbubbles with a diameter of 2.5–6.0 μm. For use, SonoVue was reconstituted in 5 ml of phosphate-buffered saline (PBS; 2–5 × 10^8^ microbubbles/ml).

The treatment protocols were as follows: a four-factor, four-level orthogonal design [L_16_(4)^4^] was employed to investigate inhibitory effects on mVEGFα expression in RM-1 cells. The parameters included the sound intensity, frequency, microbubble volume and treatment time. The levels of each parameter are reported in Table 1.

**Table 1:** The experimental design based on L_16_ (4^4^) orthogonal array and experimental results.

Number	Frequency (kHz)	Sound intensity (mW/cm^2^)	Irradiation time (s)	MB/suspension volume ratio (%)	mVEGFα expression (%)
Control group	**——**	**——**	**——**	——	100
1	20	100	30	10	76.47
2	20	180	60	20	61.27
3	20	270	90	30	83.33
4	20	360	120	40	74.51
5	80	100	60	30	84.80
6	80	180	30	40	47.55
7	80	270	120	10	35.39
8	80	360	90	20	34.61
9	500	100	90	40	44.56
10	500	180	120	30	44.26
11	500	270	30	20	30.15
12	500	360	60	10	36.03
13	800	100	120	20	41.24
14	800	180	90	10	30.15
15	800	270	60	40	45.00
16	800	360	30	30	26.52
K1	73.895	61.767	45.172	44.510	
K2	50.588	45.807	56.775	41.818	
K3	38.750	48.468	48.163	59.727	
K4	35.727	42.918	48.850	52.905	
Rvalue	38.168	18.849	11.603	17.909	

K, Sum of mVEGFα for the factors at each level; k, The mean values for the factors at each level; R, kmax-kmin.

### Detection of RM-1 mVEGFα expression via RNA extraction and reverse transcription-quantitative polymerase chain reaction (RT-qPCR)

Exponential-growth cells were digested with trypsin and collected, and the cells were counted and adjusted to obtain a density of 10^5^ cells/ml. Total RNA was isolated using TRIzol reagent (Invitrogen, USA) according to the manufacturer’s instructions. Reverse transcription was conducted according to the instructions of the RT Superscript III kit (Applied Biosystems, Inc, Carlsbad, CA, USA); the cDNA template was obtained and quantified using a Gene QuantRNA/DNA spectrophotometer. The samples were analyzed using an SYBR Green Fast 750 kit (Applied Biosystems, Inc, Carlsbad, CA, USA) with the pEasy-T3-mVEGFα plasmid (BIOSUNE, SHANGHAI CO. LTD, China) as the standard sample and beta-actin as the internal reference. The following DNA primers were synthesized by Invitrogen: murine mVEGF sense primer 5′-CACAGCAGATGTGAATGCAG-3′ and anti-sense primer 5′-TTTAAACCGGGATTTCTTGC-3′; mActB sense primer 5′-GGCTGTATTCCCTCCATCG-3′ and anti-sense primer 5′-CCAGTTGGTAACAATGCCATGT-3′. The qPCR reaction conditions were as follows: 95 °C for 5 min, followed by 40 cycles of 95 °C for 5 sec, 60 °C for 20 sec, and 72 °C for 25 sec. Melting curve analysis was conducted immediately after the reaction. Data processing included the Livak method, and target gene expression was assessed by the ratio of the copy number of the designated group and the standard sample using the 2^-△△Ct^ method.

Relative mVEGFa expression is presented as a percentage; the copy number of the control group (equal amounts of cells without ultrasound treatment and microbubbles (UM)) was recorded as 100%. According to our orthogonal experimental design and the RT-qPCR results, the level at Kmin was the best for inhibiting mVEGFa expression. Thus, the optimum parameters were frequency 800 kHz, I_SATA_ 360 mW/cm^2^, exposure time 30 sec, and a microbubble/cell suspension volume ratio of 20% (Table 1).

### Identification RM-1 cell membrane damage via Trypan blue staining

**Figure 1:**
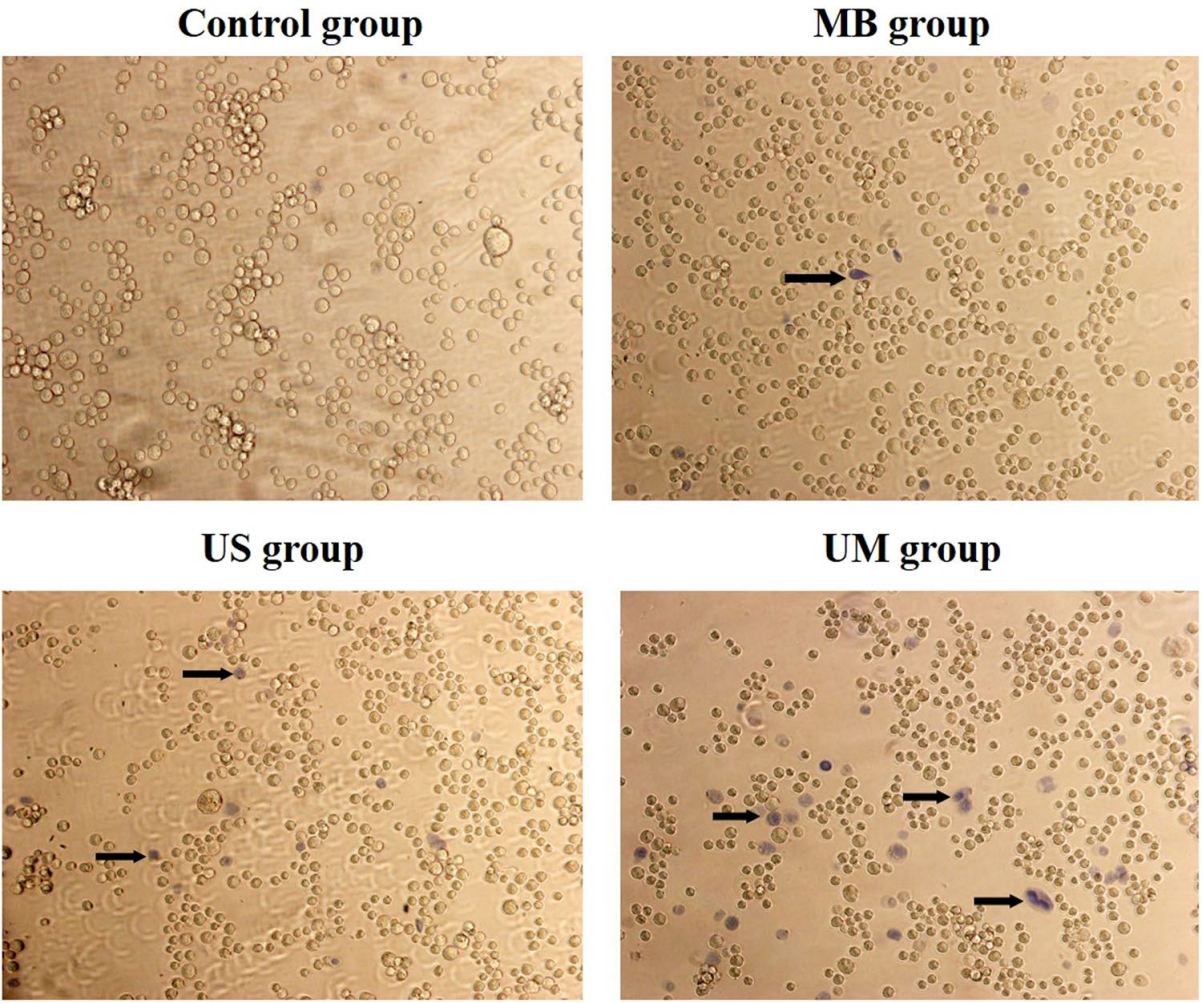
The proportion of membrane-damaged cells (labeled with black arrow) in the UM group was most obvious of all groups. The US group displayed a certain amount of blue-stained cells as well, whereas the control group and MB group showed scant staining (×200).

Membrane damage from UM was identified by Trypan blue staining. Following trypsin digestion and resuspension at 10^5^ cells/ml, RM-1 cells were divided into four groups: a control group, ultrasound group (US group), microbubble group (MB group), and ultrasound microbubble group (UM group). The cells in the US group were treated according as described above but without the addition of SonoVue. The cell suspension in the MB group was mixed with SonoVue to produce a microbubble/cell suspension volume ratio of 20% but without ultrasound treatment. The UM group included both of the abovementioned procedures, and the control group did not include either procedure. Following treatment, 0.4% Trypan blue dye was immediately added to each group; the cells were stained for 20 min, washed with PBS, and observed under an optical microscope. Membrane damage was indicated movement of the dye through the cells, causing them to appear blue (Fig. 1 and Table 2).

**Table 2:** Typan blue staining and western bolt result in each group.

Group	Proportion of blue stained cells(%)	VEGF amount by western bolt(%)
Control group	2.8 ± 0.8^a^	74.2 ± 4.7^a^
MB group	3.1 ± 1.0^a^	71.7 ± 6.6^a^
US group	10.8 ± 1.3^b^	47.9 ± 5.9^b^
UM group	19.6 ± 1.9^c^	32.7 ± 4.9^c^

^a^Compared with US group and UM group, ^b^compared with control group, MB group, and UM group, ^c^compared with control group, MB group, and US group (P < 0.05).

### VEGF expression level assessed by western blotting

**Figure 2:**
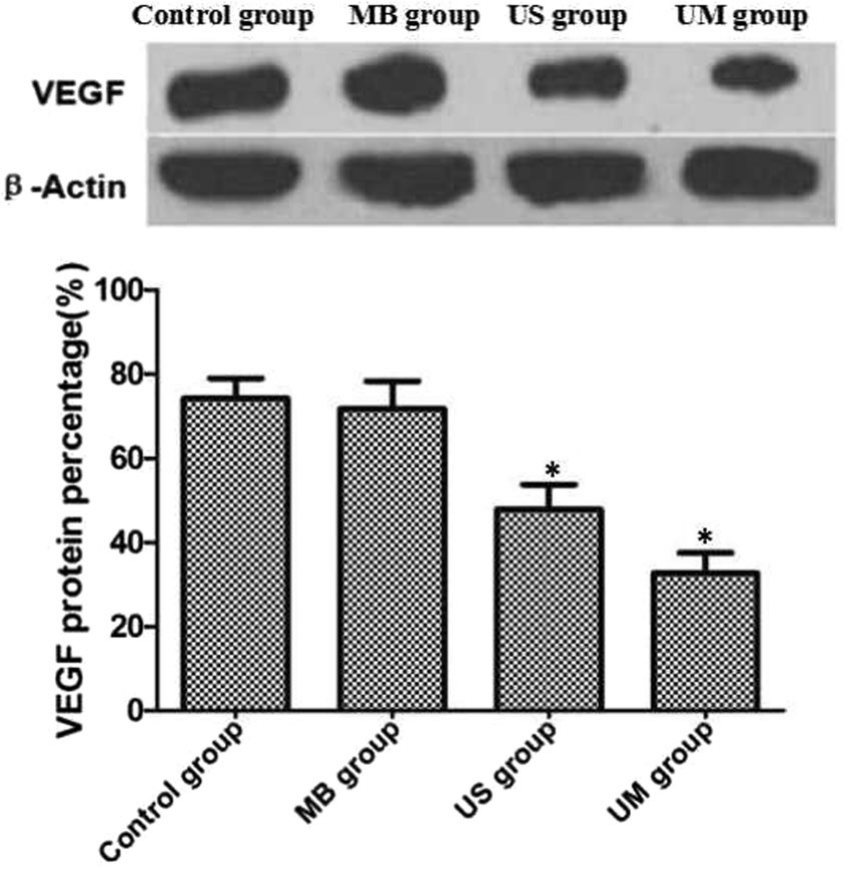
The least amount of VEGF expression was in the UM group; that of the US group was less than that of the control and MB groups (*P < 0.05 compared with other groups).

The four groups of RM-1 cells were collected at 24 h after treatment, washed with PBS and added to NP-40 buffer. The samples were centrifuged at 1,264 × g for 20 min. The supernatant was removed, loading buffer was added, and the solution was boiled for 10 min; the samples were then centrifuged at 1242 × g for 5 min. The supernatant was used for sodium dodecyl sulfate-polyacrylamide gel electrophoresis (SDS-PAGE). The separated proteins were transferred onto a nitrocellulose (NC) membrane, incubated with 5% bovine serum albumin (BSA)/PBST (PBS + Tween-20) for 30 min, and incubated overnight at 4 °C with a diluted anti-murine VEGF antibody (1:2000; Peprotech, NJ, USA). The samples were washed 3 times with PBST for 5 min each and incubated with an anti-rabbit HRP-labeled secondary antibody (1:2000; ZSGB-BIO, Beijing, China) at room temperature for 1 h. Horseradish peroxidase (HRP) was added for detection. The experiment was repeated 6 times for each group. β-Actin served as the internal reference, and the expression level of VEGF was analyzed using ImageJ software (Fig. 2 and Table 2).

### Acquisition of murine bone marrow-derived DCs and spleen-derived T lymphocytes

BALB/c mice, 6 or 8 weeks old, were obtained from SHANGHAI SLAC LABORATORY ANIMAL CO. LTD (Shanghai, China). The animals were maintained at the Central Animal Facility of Shanghai Jiao Tong University Affiliated 6th People’s Hospital according to the standard guidelines. Experiments were conducted according to the guidelines of the China Council for Animal Care.

Murine bone marrow-derived DCs were obtained from the femurs and humeri, and T lymph cells were obtained from the spleens with the addition of cytokines; the specific procedures are provided in the supplementary information.

### Detection of DC and T lymphocyte phenotypes by flow cytometry (FCM) and co-culture with RM-1 cells

#### Detection of DC phenotypes by FCM

**Figure 3:**
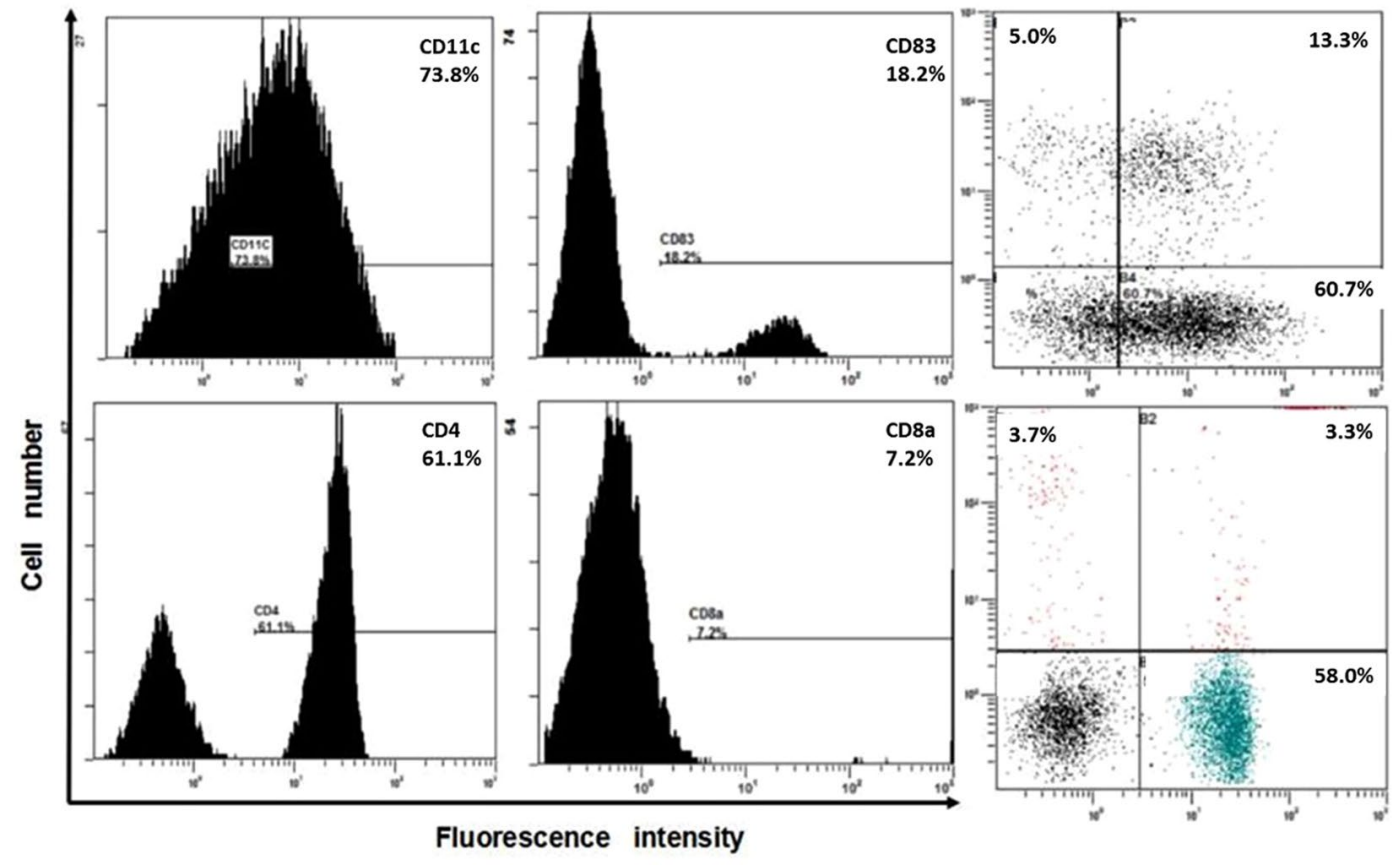
Flow cytometry detection of the phenotypes of DCs and T lymphocytes before co-culture.

DC phenotypes were confirmed by flow cytometry. Cell suspensions were washed with PBS after 48 h of co-culture, and the cells were resuspended in PBS at a density of 10^6^ cells/ml. CD11c and CD83 are surface antigens of DCs and mature DCs^19, 20^. Fluorescein isothiocyanate (FITC)-labeled anti-CD11c and PE-labeled anti-CD83 (BioLegend Inc., San Diego, USA) solutions were added to the suspensions according to the manufacturer’s instructions. Following incubation for 20 min in complete darkness at room temperature, 2 ml of PBS was added, and the tubes were centrifuged for 5 min at 158 × g. The cells were immediately analyzed using a FACScan flow cytometer (BD FACS Aria, BD Biosciences, CA, USA) following resuspension in PBS (Fig. 3).

#### Detection of T lymphocyte phenotypes by FCM

**Figure 4:**
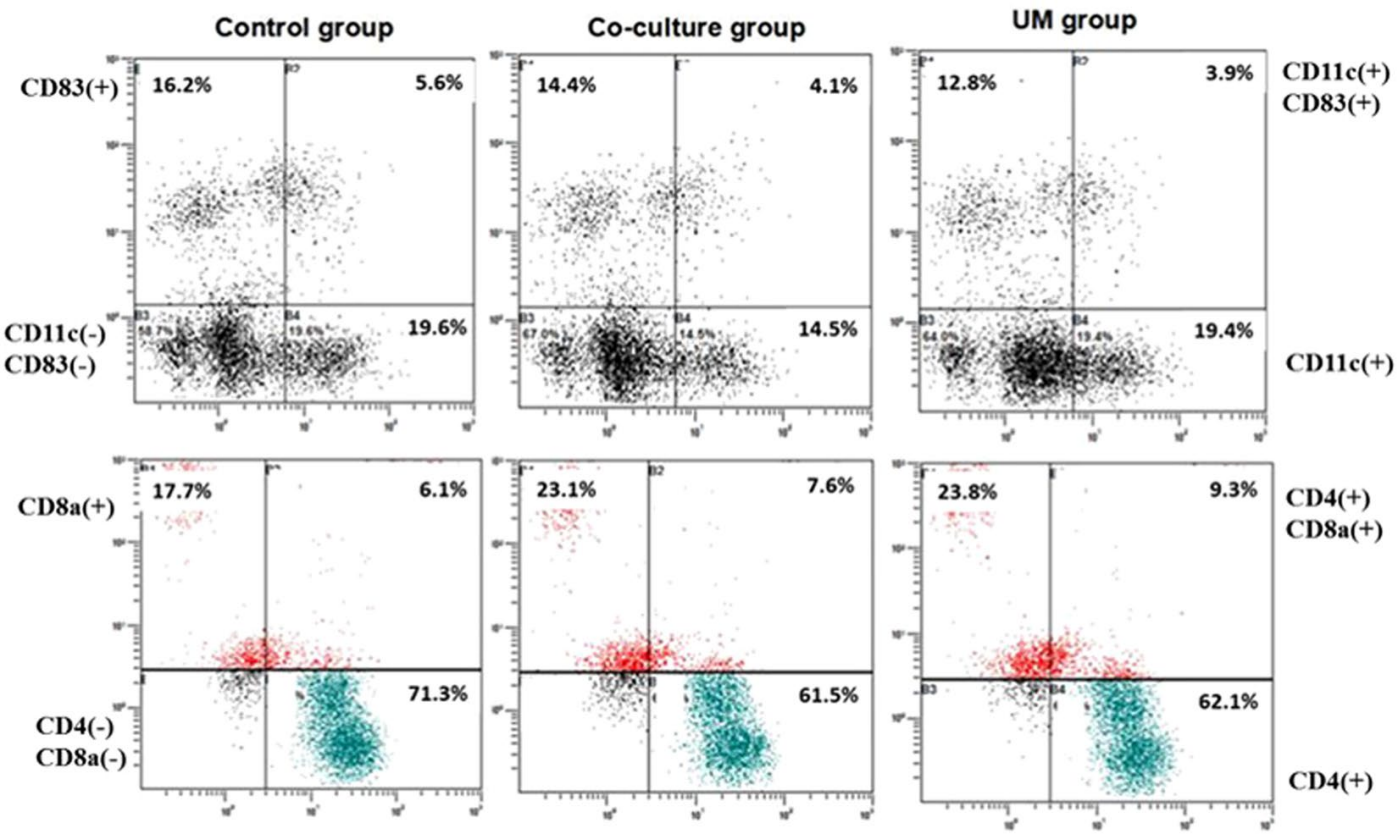
Flow cytometry detection of the phenotypes of immune cells in the control group, co-culture group and UM group after co-culture with RM-1 cells.

Following cultivation for 24 h after extraction, T lymphocytes were collected, washed with PBS and resuspended in PBS at a density of 10^6^ cells/ml. CD4(+) and CD8α(+) T lymphocytes are considered closely related to anti-tumor immunity and immune regulation in the PCa microenvironment^21^. Fluorescein isothiocyanate (FITC)-labeled anti-murine CD4 and PE-labeled anti-murine CD8α (BioLegend Inc., San Diego, USA) solutions were added to the suspensions according to the manufacturer’s instructions. The incubation and analytical methods were the same as described above for DCs (Fig. 4).

#### DCs and T lymphocytes co-culture with RM-1 cells and immunocyte phenotype detection by FCM

To determine the impact of RM-1 cells on the differentiation of DCs and T lymphocytes, DCs were combined with T lymphocytes and divided into 3 groups on the fifth day after extraction: the control group contained equal amounts of DCs and T lymphocytes; the co-culture and UM groups included DCs and T lymphocytes co-cultured with RM-1 cells (approximately 1:1:1). Transwell devices (Corning Incorporated, NY, USA) with 0.4-µm diameter holes were applied to distinguish DCs and T lymphocytes in the superstratum and RM-1 cells in the substratum. The 0.4-µm holes permitted mixing of the culture media, but the cells could not move through the holes. The co-culture methods for the two groups were identical, but the RM-1 cells in the UM group were treated with UM as described above before being co-cultured with DCs and T lymphocytes after 24 h. All cells were cultured in 6-well Costar plates, and the procedure for each group was repeated 6 times. Each group was co-cultured for 48 h before phenotypic detection by FCM (Table 3).

**Table 3:** The proportion of CD11c(+),CD83(+) DC and CD4(+), CD8a(+) T lymphocytes examined by flow cytometry (mean ± SD).

Group	CD11c(+)DC(%)	CD83(+)DC (%)	CD4(+)T lymphocytes(%)	CD8a(+)T lymphocytes(%)
Control group	24.5 ± 1.1^a^	22.3 ± 1.6^a^	74.4 ± 1.8^a^	24.3 ± 1.4^a^
Co-culture group	18.1 ± 0.4^b^	17.7 ± 2.0^b^	66.4 ± 2.3^b^	31.5 ± 0.8^b^
UM group	22.2 ± 0.9^c^	16.9 ± 0.7^b^	67.7 ± 1.6^b^	34.0 ± 1.5^c^

^a^Compared with co-culture group and UM group, ^b^compared with control group and UM group, ^c^compared with control group and co-culture group (P < 0.05).

### Exploring the synergistic effect of low-frequency ultrasound and immunocytes on RM-1 cell inhibition *in vitro*

To determine the influence of DCs and T lymphocytes on RM-1 cells, lymphocytes were collected and divided into four groups. The control group contained RM-1 cells without treatment. The ultrasound group was treated with low-frequency ultrasound according to the method described above following the addition of SonoVue. The immunization group contained RM-1 cells, DCs and lymphocytes in a 1:1:1 ratio, and the ultrasound+immunization group (UI group) included equal amounts of ultrasound-treated RM-1 cells co-cultured with DCs and lymphocytes. All groups were cultured in 6-well Costar plates for 48 h after treatment.

#### Observation of cell migration using a scratch assay

**Figure 5:**
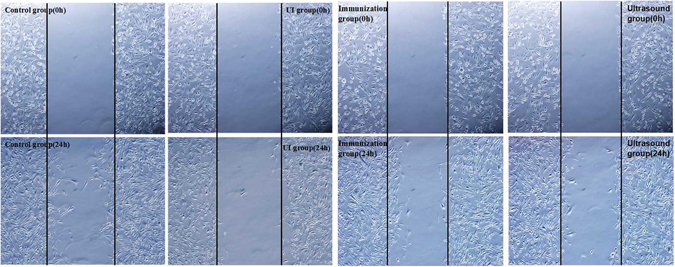
RM-1 cells in the control group has dispersed into the scratch area. Cell migration by the UI group was not obvious; a certain number of cells crossed the line in the immunization group and ultrasound group (×100).

Tumor cell migration was assessed with a scratch assay as previously described. In brief, 0.5–1.0 × 10^6^ cells were seeded onto coated 60-mm dishes and incubated for 6 h. The supernatant was discarded, and the cells were washed 3 times with PBS. Two parallel lines were drawn on the bottom of each well, and 3 lines were scratched perpendicular to the former lines using a P200 pipette tip. Displaced cells were carefully removed by washing with PBS, and the remaining cells were cultured with low-FBS (2% FBS) RPMI-1640 and allowed to migrate for the indicated times. Photographs were obtained at 0 and 24 h (Fig. 5). Six views were randomly chosen, and cell numbers between the edges of the scratch were measured to quantitatively evaluate cell migration.

#### Cytoactive detection using a cell CCK-8 absorbency test

RM-1 cells of all groups were digested and collected after cultivation for 48 h, followed by centrifugation and resuspension to obtain 10^5^ cells/ml. A 100-µl volume of inoculated cell suspension was placed in each well of a 96-well Costar plate (Corning Incorporated, NY, USA); each group was repeated in 10 wells. A 10-µl volume of CCK-8 solution was carefully added to each peripheral well to avoid air bubble influence on the O.D. value. The Costar plate was placed in an incubator for 3 h, and absorbance at 450 nm was determined using a Tecan Sunrise microplate reader. The results are presented as the O.D. value (Fig. 7 and Table 4).

**Table 4:** Cell num ber in the scratched and transwell area and O.D value in each group.

Group	Cell number in the scratched area	O.D value	Cell number after transwell experiment
Control group	122 ± 6.5^a^	0.589 ± 0.071^a^	252 ± 15.2^a^
Ultrasound group	69 ± 8.1^b^	0.232 ± 0.031^b^	179 ± 18.8^b^
Immunization group	76 ± 6.9^b^	0.270 ± 0.042^c^	191 ± 12.1^b^
UI group	44 ± 4.7^c^	0.202 ± 0.028^d^	105 ± 14.9^c^

(Statistical index labeled with different characters P < 0.05).

#### Cell invasive ability assessed by Transwell experiments

RM-1 cells of all groups were collected after digestion, centrifuged and resuspended at 10^6^ cells/ml in FBS-free RPMI-1640. The samples were cultured for 24 h and resuspended with RPMI-1640 containing 0.1% BSA (Biosharp, Hefei China) following digestion and centrifugation.

**Figure 6:**
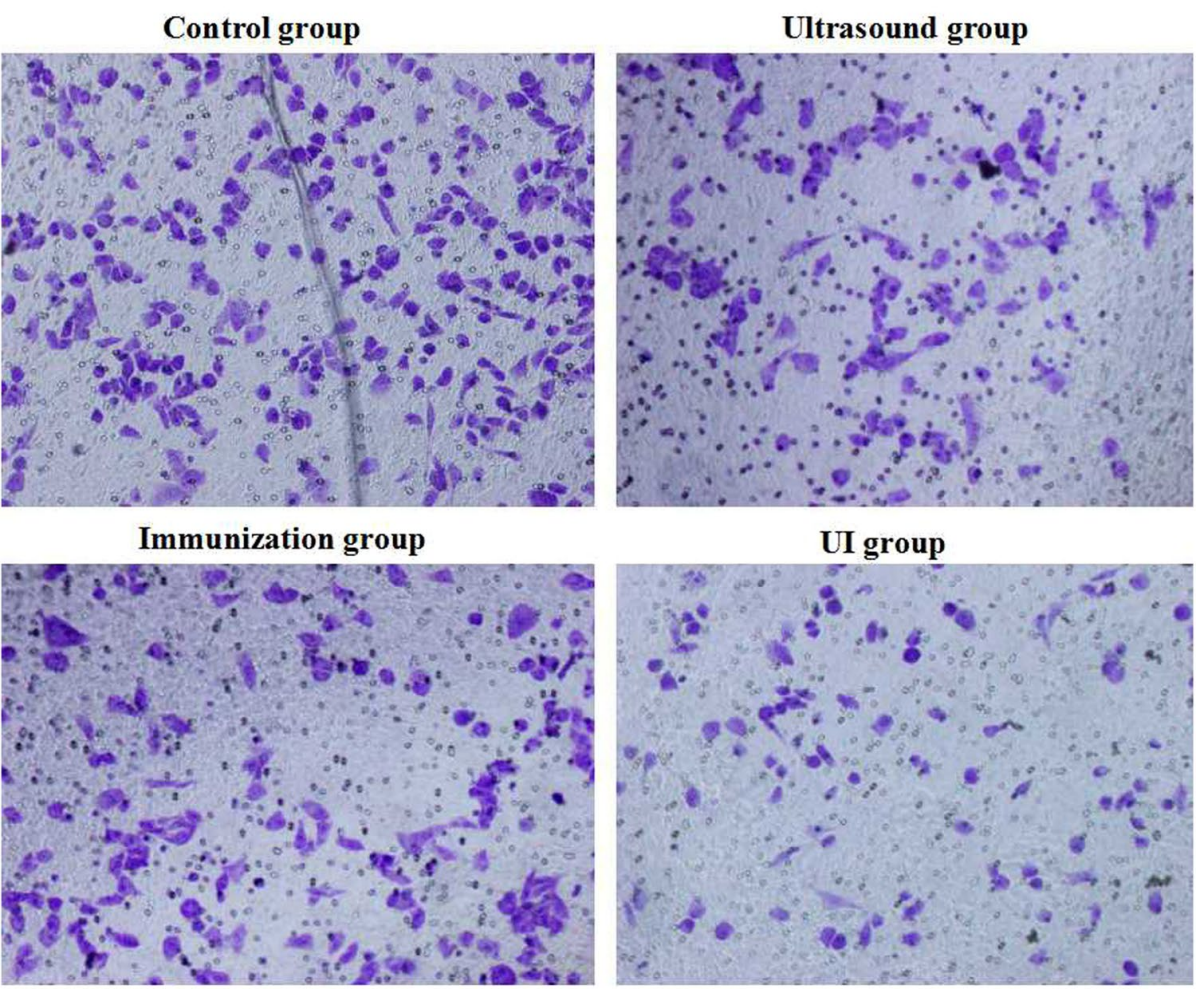
Numerous cells passed through the Matrigel matrix in the control group; fewer cells of the immunization and ultrasound groups invaded; cell invasiveness in the UI group was the weakest (×200).

Matrigel matrix basement membrane (Corning Incorporated, NY, USA) was diluted at a 1:8 ratio using FBS-free RPMI-1640. Transwell devices (Corning Incorporated, NY, USA) with an 8.0-µm pore size and 6.5-mm diameter inserts were used with a 24-well Costar plate. Diluted Matrigel matrix (70 µl) was added to the superstratum of the Transwell on ice to ensure that the matrix remained in a liquid state. The Transwell devices were placed in an incubator, and superfluous culture solution was absorbed 30 min later. Then, 150 µl of cell suspension was added to the superstratum, and 500 µl of RPMI-1640 with 10% FBS was added to the substratum. The samples were cultured for 24 h, and the cells in the superstratum were gently wiped off; the cells on the undersurface were visualized using crystal violet (Beyotime, Jiangsu, China) staining. The outcome is represented by the cell number of 5 regions randomly selected per group (Fig. 6).

### Statistical analysis

Trypan blue staining was analyzed using five fields randomly selected for each group, and the data are presented as the proportion of blue stained cells (mean ± SD). Images of the western blot and scratch assays were evaluated using the semi-quantitative software ImageJ. Analysis of variance (ANOVA) was used to evaluate the results of flow cytometry and the absorbency test O.D. values of all groups. Inter-group comparisons were performed using the SNK-q test (equal variances assumed) or Dunnett’s C test (equal variances not assumed). The positive rate is presented as the mean ± SD, and P < 0.05 was considered statistically significant.

## Results

### Trypan blue staining observed by optical microscopy

Five fields were selected per group, and the results are presented as the proportion of blue-stained cells. Few cells were dyed blue in the control group (2.8 ± 0.8%) or the MB group (3.1 ± 1.0%), indicating that cavitation resulting in membrane damage hardly occurred without low frequency ultrasound. For the US group, blue staining was observed in 10.8% of cells, suggesting that the culture solution contained few microbubbles and that the amount of ultrasound treatment was inadequate, with only mild cavitation. The greatest amount of membrane damage was found for the UM group (19.6 ± 1.9%); cavitation in this group was vigorous with the application of both ultrasound and microbubbles (Fig. 1 and Table 2).

### VEGF expression via western blotting

For intuitive comparison, VEGF protein expression was compared proportionally. β-Actin served as the internal reference, and the standard sample was defined as 100%. Cavitation in the UM group was the most obvious, and the least VEGF expression was observed in this group (32.7 ± 4.9%). Mild cavitation in US group was accompanied by suppression of VEGF protein expression (47.9 ± 5.9%). In contrast, the control group and MB group appeared to be unaffected, with no significant difference between them (Fig. 2 and Table 2)

### FCM detection of DC and T lymphocyte phenotypes

The FCM results of DCs were determined before co-culture (on the fifth day after extraction). The proportions of CD11c(+) and CD83(+) cells were 73.8% and 18.2%, respectively, which indicated that the system contained specific amounts of DCs and mature DCs. Moreover, the proportions of CD4(+) and CD8a(+) T lymphocytes were 61.1% and 7.2%, respectively, also indicating that the system contained specific amounts of T lymphocytes and CTLs (Fig. 3).

The abundance of CD11c(+)/CD83(+) DCs and CD4(+)/CD8a(+)T lymphocytes in the control group was higher than in the other groups, which indicated that immune cell differentiation was suppressed in the PCa microenvironment. The proportion of CD11c(+) DCs and CD8a(+) T cells in the UM group was higher than that of the co-culture group, further showing that inhibition of VEGF expression in RM-1 cells using ultrasound and microbubbles could promote DC and CTL proliferation in the co-culture system (Fig. 4 and Table 3).

### Observation of cell migration via the scratch assay

In the scratch test, the UI group showed the fewest cells passing through the membrane, indicating the weakest migration ability. For ultrasound group and immunization group, the number of migrating cells was lower than that in the control group, indicating that ultrasound and anti-tumor immunity can inhibit cell migration (Fig. 5 and Table 4).

### Cell invasive ability assessed via Transwell experiments

Few cells passed through the matrix in the UI group, indicating that the invasive ability of this group was the weakest. The invasive capacities of the ultrasound and immunization groups were less than that of the control group. These results show that ultrasound and anti-tumor immunity can inhibit RM-1 invasion (Fig. 6 and Table 4).

### Cytoactive detection using a cell CCK-8 absorbency test

**Figure 7:**
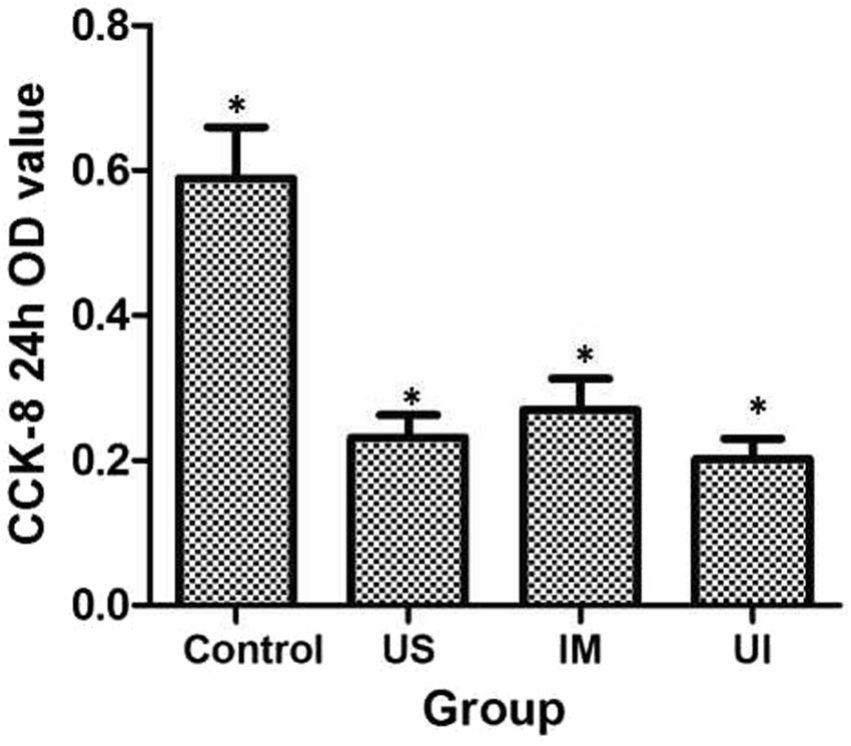
Absorbance at 450 nm, indicating proliferation capacity according to the CCK-8 test, was weakest in the UI group (*P < 0.05 compared with other groups).

The O.D. value at 450 nm, which represents the proliferation ability of RM-1 cells, was determined using a Tecan Sunrise microplate reader at 3 h after treatment. The sequence from high to low proliferation was as follows: the control group, ultrasound group, immunization group and UI group (Fig. 7 and Table 4).

## Discussion

Up-regulation of both angiogenesis and immunodeficiency is closely related to tumorigenesis and progression. Therefore, targeting angiogenesis and immunoregulation is a promising modality in oncotherapy. Low-frequency ultrasound (frequency between 20 kHz and 1 MHz) may induce cavitation via destructing microbubbles (MBs) in liquid. These bubbles may serve as targets, resulting in local release of energy in the form of radiation force, microstreaming, shock waves, free radicals, microjets and strain^22^. This energy may further trigger a series of biological effects, including vascular endothelial injury and protein denaturation. In general, cavitation in an ultrasound field increases with decreasing frequency and increasing MB density^23^. For example, Wang^24^ reported that the chemotherapeutic effect was enhanced in PCa cells when the low-frequency ultrasonic density was fixed at 113 mw/cm^2^, which likely occurred via alterations in membrane integrity and permeability. Furthermore, it has been reported that the survival rate of tumor cells decreases with increasing ultrasound intensity and duration^25^. As the MB density in tissue is typically low, exogenous MBs, such as SonoVue, are required to increase cavitation in the tumor microenvironment; nonetheless, sonoporation stagnates when MBs reach a certain concentration. In the present study, we adopted the optimized parameter of low-frequency ultrasound at 800 kHz, ultrasonic power of 360 mW/cm^2^, and a microbubble/cell suspension volume ratio of 20% to significantly inhibit VEGF expression in RM-1 cells. In this case, the greater intensity directly induced irreversible membrane damage and apoptosis. Moreover, the low frequency and microbubbles generated inertial cavitation, resulting in the fragmentation and destruction of the fragile cellular structure of the cancer cells^26^.

It has been reported that immunocyte differentiation is negatively correlated with VEGF-expressing tumors^27^, especially in renal carcinoma, ovarian carcinoma and melanoma^28–30^. This indicates that VEGF participates in immunosuppression in the tumor microenvironment, especially with regard to DC activity, providing a theoretical basis for further research. Despite numerous studies on VEGF and DC-related immunology in tumor microenvironments, suppressing angiogenesis with ultrasound and simultaneously activating anti-tumor immunity has seldom been mentioned. Thus, our study is innovative in this field.

In this study, all DCs and lymphocytes were directly obtained from the bone marrow and spleens of the same BALB/c mice. Moreover, cytokine stimulation of all co-cultures began on the 5th day, which ensured that the initial immunological states were identical and that inter-individual immunological interference was reduced to a minimum. For detection after US combined with MBs, we used Trypan blue staining and western blot analysis; the former approach provided visual, macroscopic and qualitative information, whereas the latter provided objective, microcosmic and quantitative information. In the detection of DC and T lymphocyte phenotypes, flow cytometry not only verified these immune cells in the system but also contributed to tracking variation tendency. The scratch assay, CCK-8 and Transwell experiments comprise three simple and convenient methods for detecting RM-1 cell migration, proliferation and invasion capacities, respectively. These approaches allowed us to readily draw conclusions based on differences between the groups.

Both mature and immature DCs express the surface antigen CD11c; however, expression tends to decrease during maturation. Moreover, DCs were further diluted with the addition of T lymphocytes. This may help to illustrate why the proportion of CD11c(+) DCs was significantly decreased in all groups following co-culture. Furthermore, CD83 was only expressed on the surface of extracorporeally stimulated mature DCs, and T lymphocytes were activated by DCs and cytokines in the PCa microenvironment. Thus, we speculate that despite mixing, the gross increase in mature DCs and T lymphocytes caused the detection rates of CD83(+) DCs and CD4(+) and CD8(+) T lymphocytes to increase somewhat. The scratch test revealed greater inhibition of tumor migration for the UM group than the immunization group. Combining the literature with our experimental results, we suggest that UM treatment had direct serious biological effects (mechanical, thermal, chemical and cavitation) on the cells *in vitro* and that the culture suspension contained soluble microbubbles, promoting mild cavitation. As the immunologic state *in vitro* is not identical to that *in vivo*, with the type and amount of cytokines and immunocytes differing, anti-tumor immunity might be weaker than that in the intracorporal condition.

There are several limitations to our work. One limitation is that the study was completely conducted *in vitro*, which cannot completely mimic the microenvironment *in vivo*. Another limitation may be that in the mixture of cells extracted, sophisticated interference by various cell types may have imposed restrictions on further analysis of the mechanism involved in immunological modification. Thus, future research should utilize improved methods for the isolation and purification of DCs and T lymphocytes.

## Conclusion

Low-frequency ultrasound combined with microbubbles significantly inhibits VEGF expression in murine prostate cancer RM-1 cells and simultaneously promotes DC differentiation in the extracorporeal prostate cancer microenvironment. These conditions prompted DCs and T lymphocytes, especially immature DCs and CD8(+) CTLs, to further trigger T lymphocyte-mediated anti-tumor immunity, enhancing the efficacy of angiogenesis targeting.

## Electronic supplementary material

Supplementary information
