# Supplementary material for: Low-frequency ultrasound-induced VEGF suppression and synergy with dendritic cell-mediated anti-tumor immunity in murine prostate cancer cells in vitro
Source: Sci Rep. 2017 Jul 18;7:5778. doi: 10.1038/s41598-017-06242-8 (PMC5515892; doi:10.1038/s41598-017-06242-8)
Supplement: Supplementary file 1 — Supplementary information [file 41598_2017_6242_MOESM1_ESM.doc]

**Low-frequency ultrasound-induced VEGF suppression and the synergy with dendritic cell-mediated anti-tumor immunity in murine prostate cancer cells in vitro**

WEI ZHANG, WEN-DE SHOU,YAN-JUN XU，WEN-KUN BAI**^※^**, and BING HU**^※^**

Department of Ultrasound In Medicine, Shanghai Jiao Tong University Affiliated 6th People’s Hospital, Shanghai Institute of Ultrasound in Medicine, Shanghai, China, 200233

**^※^**Corresponding author:

Wenkun Bai, Shanghai Jiao Tong University Affiliated sixth People’s Hospital, Department of Ultrasound In Medicine, Shanghai Institute of Ultrasound In Medicine, Yishan Road 600, Shanghai, China 200233. Telephone number: +86 21 64369181x8877, Fax number: +86 21 54488254. E-mail:doctor[505@hotmail.com;](mailto:505@hotmail.com;)

Bing Hu, Shanghai Jiao Tong University Affiliated sixth People’s Hospital, Department of Ultrasound In Medicine, Shanghai Institute of Ultrasound In Medicine, Yishan Road 600, Shanghai, China 200233. Telephone number: +86 21 64369181x8877, Fax number: +86 21 54488254. E-mail:hubing_us@163.com

**Murine bone marrow-derived DCs were obtained from the femurs and humeri of Balb/c mice as follows:**

1. The cervical vertebrae were removed, and the body was placed in rubbing alcohol for 10 min.

2. The femurs and humeri were dissociated in a clean petri dish that contained 5 ml of RPMI-1640 without FBS.

3. The two ends of the bones were cut off, and the medullary cavities were flushed until they turned white.

4. The cell suspension was collected and centrifuged for 5 min at 158 x g; the supernatant was subsequently discarded, and 3 ml of red cell lysis buffer was added （TIANGEN BIOTECH, BEIJING CO. LTD, China).

5. After 3 min, 15 ml of RPMI-1640 was added, and the samples were centrifuged again.

6. The cells were resuspended and counted to obtain a density of 105 cells/ml. The cytokines murine GM-CSF and IL-4 (PEPROTECH, NJ, USA) were added in final concentrations of 20 ng/ml and 10 ng/ml, respectively.

7. The cells were incubated at 37°C and in 5% CO2 to allow for their differentiation into DCs; RPMI-1640 was replaced and the cytokines were replenished every 72 h.

**T lymph cells were obtained from the spleens of Balb/c mice as follows:**

1. The cervical vertebrae were removed, and the body was placed in rubbing alcohol for 10 min.

2. The spleen was dissociated and cut into small pieces with a diameter of 2-3 mm.

3. The pieces were ground on a 200-mesh sieve and washed with PBS; 3 ml of solution was obtained.

4. The solution was collected and slowly added to the surface layer of 6 ml of mouse lymphocyte separation medium (DAKEWE, BIOTECH, SHENZHEN, CHINA).

5. The samples were centrifuged for 20 min at 211 x g; the lymphocyte layer was slowly absorbed and washed with PBS.

6. The samples were centrifuged for 5 min at 158 x g; the supernatant was discarded, and the samples were resuspended with 2 ml of RPMI-1640 that contained 10% FBS.

7. A nylon wool fiber column with 500 mg in a 10-ml injector, which was connected with a Tee-junction, was vertically placed on a brandreth; 20 ml of RPMI-1640 (FBS free) and 15 ml of RPMI-1640 (10% FBS) were successively added and removed, and the valve was turned off.

8. The samples were added to lymphocyte buffer and 1 ml of RPMI-1640 (10% FBS); the valve was turned on, and 1 ml of liquid was released. The valve was then turned off again.

9. The device was wrapped with tinfoil and placed in an incubator for 60 min; the injector was vertical during the entire procedure.

10. The tinfoil was removed, and the valve was placed in rubbing alcohol for 5 min. A 2-ml volume of RPMI-1640 (10% FBS) was then added; the valve was turned on, and all of the solution was released at a rate of 1 drop/sec.

11. The solution was collected, centrifuged for 5 min at 158 x g, and resuspended with 4 ml of RPMI-1640 (10% FBS).
